# Polymerase II–Associated Factor 1 Complex-Regulated *FLOWERING LOCUS C*-Clade Genes Repress Flowering in Response to Chilling

**DOI:** 10.3389/fpls.2022.817356

**Published:** 2022-02-09

**Authors:** Zeeshan Nasim, Hendry Susila, Suhyun Jin, Geummin Youn, Ji Hoon Ahn

**Affiliations:** Department of Life Sciences, Korea University, Seoul, South Korea

**Keywords:** *Arabidopsis*, FLC, MAFs, PAF1C, flowering, epigenetics, temperature

## Abstract

RNA polymerase II–associated factor 1 complex (PAF1C) regulates the transition from the vegetative to the reproductive phase primarily by modulating the expression of *FLOWERING LOCUS C* (*FLC*) and *FLOWERING LOCUS M* [*FLM*, also known as *MADS AFFECTING FLOWERING1* (*MAF1*)] at standard growth temperatures. However, the role of PAF1C in the regulation of flowering time at chilling temperatures (i.e., cold temperatures that are above freezing) and whether PAF1C affects other *FLC*-clade genes (*MAF2*–*MAF5*) remains unknown. Here, we showed that *Arabidopsis thaliana* mutants of any of the six known genes that encode components of PAF1C [*CELL DIVISION CYCLE73/PLANT HOMOLOGOUS TO PARAFIBROMIN*, *VERNALIZATION INDEPENDENCE2* (*VIP2*)/*EARLY FLOWERING7* (*ELF7*), *VIP3*, *VIP4*, *VIP5*, and *VIP6/ELF8*] showed temperature-insensitive early flowering across a broad temperature range (10°C–27°C). Flowering of PAF1C-deficient mutants at 10°C was even earlier than that in *flc*, *flm*, and *flc flm* mutants, suggesting that PAF1C regulates additional factors. Indeed, RNA sequencing (RNA-Seq) of PAF1C-deficient mutants revealed downregulation of *MAF2–MAF5* in addition to *FLC* and *FLM* at both 10 and 23°C. Consistent with the reduced expression of *FLC* and the *FLC*-clade members *FLM/MAF1* and *MAF2–MAF5*, chromatin immunoprecipitation (ChIP)-quantitative PCR assays showed reduced levels of the permissive epigenetic modification H3K4me3/H3K36me3 and increased levels of the repressive modification H3K27me3 at their chromatin. Knocking down *MAF2–MAF5* using artificial microRNAs (amiRNAs) in the *flc flm* background (*35S::amiR-MAF2–5 flc flm*) resulted in significantly earlier flowering than *flc flm* mutants and even earlier than *short vegetative phase* (*svp*) mutants at 10°C. Wild-type seedlings showed higher accumulation of *FLC* and *FLC*-clade gene transcripts at 10°C compared to 23°C. Our yeast two-hybrid assays and *in vivo* co-immunoprecipitation (Co-IP) analyses revealed that MAF2–MAF5 directly interact with the prominent floral repressor SVP. Late flowering caused by *SVP* overexpression was almost completely suppressed by the *elf7* and *vip4* mutations, suggesting that SVP-mediated floral repression required a functional PAF1C. Taken together, our results showed that PAF1C regulates the transcription of *FLC* and *FLC*-clade genes to modulate temperature-responsive flowering at a broad range of temperatures and that the interaction between SVP and these FLC-clade proteins is important for floral repression.

## Introduction

Plant survival and fitness depends on timely seed production through precise control of flowering time. Flowering time is modulated by a number of endogenous and environmental cues, such as daylength, age, and prolonged exposure to cold and ambient temperatures (Amasino, 2010; Srikanth and Schmid, 2011). To successfully survive a range of varying environmental conditions, plants have evolved a complex regulatory network that integrates these cues to control flowering time (Srikanth and Schmid, 2011). In Arabidopsis (*Arabidopsis thaliana*), nearly 400 flowering genes are known to regulate flowering time in genetically distinct pathways, e.g., the photoperiod, ambient temperature, aging, vernalization, hormonal, and sugar pathways (Bernier and Périlleux, 2005; Bouché et al., 2016). These pathways modulate flowering in response to different endogenous and environmental signals to optimize reproductive success.

Among the flowering time genes, *FLOWERING LOCUS C* (*FLC*), encoding a MADS-box transcription factor, is an important repressor of flowering in various plant species, including Arabidopsis (Michaels and Amasino, 1999; Sheldon et al., 1999; Ruelens et al., 2013). In Arabidopsis winter accessions, FRIGIDA (FRI) induces *FLC* transcription, whereas vernalization (prolonged exposure to cold) epigenetically represses *FLC* transcription (Johanson et al., 2000; Choi et al., 2011). By contrast, in rapid-cycling accessions lacking functional *FRI*, members of the autonomous pathway regulate *FLC* transcription (Amasino, 2010). FLC negatively regulates flowering by directly repressing the transcription of two important genes that promote flowering, *FLOWERING LOCUS T* (*FT*), and *SUPPRESSOR OF OVEREXPRESSION OF CO1* (*SOC1*; Helliwell et al., 2006; Searle et al., 2006; Li et al., 2008).

In addition to *FLC*, the Arabidopsis genome contains five more members of the *FLC* clade, e.g., *MADS AFFECTING FLOWERING1* [*MAF1*, also known as *FLOWERING LOCUS M* (*FLM*)] and *MAF2*–*MAF5* (Ratcliffe et al., 2001, 2003; Scortecci et al., 2001). The *MAF2–MAF5* genes occur in a tandem repeat within a 22-kb region. The role of *FLM* as a floral repressor has been well-studied (Scortecci et al., 2003; Balasubramanian et al., 2006), and its loss of function causes temperature-insensitive flowering (Lee et al., 2013). Like *FLM*, *MAF2* acts as a floral repressor, and the loss of *MAF2* function results in strong acceleration of flowering upon vernalization, whereas plants overexpressing *MAF2* flowered significantly later than wild-type plants (Ratcliffe et al., 2003). Similarly, MAF3 also represses flowering by directly binding to the promoter sequences of *FT* and *SOC1* and repressing their transcription. Interestingly, the effect of the loss of *MAF3* function was more evident at lower temperatures than at normal growth temperatures (Gu et al., 2013). Overexpression of *MAF3* produced a stronger floral delay in the Landsberg *erecta* (L*er*) accession compared to Columbia (Col-0; Ratcliffe et al., 2003). T-DNA mutants of *MAF4* exhibited accelerated flowering (Gu et al., 2009), and *MAF4* overexpression in L*er* resulted in a strong delay of flowering (Scortecci et al., 2003). Unlike *MAF1–MAF4*, the floral repressive effect of *MAF5* is not strong, and its overexpression only delayed flowering under non-inductive short-day conditions (Kim and Sung, 2010). These FLC-clade transcription factors physically interact with each other, and some of them interact with SHORT VEGETATIVE PHASE (SVP) to make repressor complexes for efficient floral repression (Gu et al., 2013).

Expression of *FLC*-clade genes is epigenetically regulated by a number of histone modifiers that are recruited to these loci by Polymerase II-Associated Factor 1 Complex (PAF1C; Zhang and Van Nocker, 2002; Zhang et al., 2003; Oh et al., 2004). In Arabidopsis, PAF1C components include CELL DIVISION CYCLE73 (CDC73)/PLANT HOMOLOGOUS TO PARAFIBROMIN (PHP), VERNALIZATION INDEPENDENCE2 (VIP2)/EARLY FLOWERING7 (ELF7), VIP3, VIP4, VIP5, and VIP6/ELF8 (Zhang and Van Nocker, 2002; Zhang et al., 2003; He, 2009; Yu and Michaels, 2010). Components of PAF1C interact with RNA Polymerase II (Betz et al., 2002) and recruit H3K4 methyltransferase to their target genes, primarily in the 5′ transcribed regions, thereby leading to the active expression of their target genes (Ng et al., 2003b). In addition to methyltransferases, PAF1C interacts with the splicing factor SKI-INTERACTING PROTEIN (SKIP) to modulate expression of *FLC* and *FLM* (Cao et al., 2015; Li et al., 2019).

Genetic studies revealed that lesions in these PAF1C components resulted in nearly identical early flowering phenotypes under standard growth conditions (He et al., 2004; Oh et al., 2004). PAF1C is required for the enrichment of active epigenetic marks, primarily H3K4me3, at the chromatin of *FLC* and its homologs to maintain their expression; PAF1C deficiency results in reduced expression of these floral repressors, which eventually accelerates flowering (Zhang et al., 2003; He et al., 2004; Yu and Michaels, 2010). For instance, loss of function of *ELF7* and *VIP6* results in the reduced expression of *FLC*, *FLM*/*MAF1*, and *MAF2* (He et al., 2004). Mutations in *VIP3* strongly reduced *FLC* expression and strongly accelerated flowering. In particular, *vip3* mutants flowered significantly earlier than *flc* mutants, suggesting that additional genes are involved in the early flowering of *vip3* mutants (Zhang et al., 2003). Loss of function of *VIP4* and *VIP5* also resulted in strong flowering acceleration that was comparable to that seen in *vip3* single mutants. However, after vernalization, the H3K27 methyltransferase complex Polycomb repressive complex 2 (PRC2) is involved in silencing *FLC* expression (and probably *MAF* expression) by depositing repressive H3K27me3 marks on the *FLC* chromatin (Wood et al., 2006; De Lucia et al., 2008). Although the regulation of *FLC* (and some *MAF* genes) is well-studied under standard growth temperature conditions, whether PAF1C-mediated regulation involves the entire *FLC* clade and the functional importance of this clade in regulating flowering at chilling temperatures remain unclear.

Here, we showed that PAF1C epigenetically regulates the entire set of *FLC*-clade genes. PAF1C-defective mutants showed ambient temperature-insensitive early flowering due to the downregulation of *FLC*-clade genes. The epigenetic status of the chromatin of *FLC* and *FLC-*clade genes was altered in PAF1C-defective mutants. Expression of *FLC* and *FLC*-clade genes was upregulated in response to low temperature in wild-type plants, and these genes play an important role in floral repression at chilling temperatures. Furthermore, MAF2–MAF5 physically interacted with SVP, and SVP-mediated floral repression requires PAF1C, suggesting the possibility that larger repressive complexes form to prevent precocious flowering, especially at chilling temperatures.

## Materials and Methods

### Plant Materials and Growth Conditions

The *Arabidopsis thaliana* mutant lines for *ELF7* (*elf7-2*; SALK_ 046605 and SALK_070632; hereafter *elf7-4*), *CDC73* (*cdc73-1*; SALK_150644 and *cdc73-2*; SALK_008357), *VIP3* (*vip3-2*; SALK_083364 and *vip3-6*; SALK_060207), *VIP4* (*vip4-1*; SALK_122755 and *vip4-3*; SALK_006392), *VIP5* (*vip5-2*; SALK_062223 and SALK_055889; hereafter *vip5-3*), and *VIP6* (*vip6-2*; SALK_065364 and SALK_119910, hereafter *vip6-5*) were obtained from the ABRC. Genotyping of the mutant lines was performed using the primers described in Supplementary Table 1. The effect of the T-DNA insertion on gene expression was confirmed *via* RT-PCR for the uncharacterized mutant lines (*elf7-4*, *vip5-3*, and *vip6-5*). The *35S::SVP:HA* lines were previously generated (Cho et al., 2012). For expression analyses at different temperatures, seedlings from the identical developmental stage of 1.02 (8-day-old seedlings at 23°C and 22-day-old seedlings at 10°C; Boyes et al., 2001) grown under standard long-day (LD) conditions (16:8 h light:dark) were used. LED lights with a light intensity of 120 μmol m^−2^ s^−1^ were used in this study. Flowering time was measured as the total leaf number, and the data are presented as box plots generated using the “PlotsOfData” app in the R package shiny (Postma and Goedhart, 2019), with customized settings.

### RNA Sequencing

RNA sequencing (RNA-Seq) was performed using 8-day-old seedlings grown at 23°C and 23-day-old seedlings grown at 10°C under standard LD conditions in two biological replicates for each sample. About 60–80 seedlings were harvested at Zeitgeber Time 16 (ZT16) and pooled for RNA extraction using Invitrogen’s Plant RNA Purification Reagent. For RNA sequencing, library preparation was performed with an Illumina TruSeq Stranded Total RNA Sample Prep kit (Illumina), according to the manufacturer’s protocols, and paired-end reads were produced using an Illumina HiSeq2000 sequencer. The raw RNA-seq data of PAF1C-deficient mutants generated in this study were deposited at the Gene Expression Omnibus (GEO) NCBI database and are available under the accession number GSE171778. Transcriptome data for *sdg8* mutants (GEO accession number GSE8528) were previously published (Pajoro et al., 2017).

### RNA-Seq Data Analyses

The raw sequence reads were processed by adapter trimming, followed by qualitative analysis of raw reads using FastQC.^1^ The resulting good quality reads were aligned to the TAIR10 reference genome using CLC Genomics Workbench v.11. Differentially expressed genes (DEGs) were defined as genes with at least a 1.5-fold change, unless mentioned otherwise. Heatmaps were generated using the built-in function of CLC Genomics Workbench. Gene Ontology (GO) analysis was performed with DAVID (Ashburner et al., 2000), and GO enrichment data plotting was performed using the R package ggplot2 (Wickham, 2011). For the identification of common targets, the intersection of the gene lists was identified using the R package UpSetR (Lex et al., 2014) and the Java-based program VennDis (Ignatchenko et al., 2015).

### Reverse Transcription Quantitative PCR Analyses

Reverse transcription quantitative PCR (RT-qPCR) was used to validate the RNA-seq data obtained from the PAF1C-deficient mutants. Total RNA was extracted from seedlings at the identical developmental stage at ZT16 at different temperatures. Plant RNA purification reagent (Invitrogen) was used for RNA extraction. The DNase I–treated RNA (~2 μg) was reverse transcribed into cDNA using MMLV enzyme (ELPIS Biotech). qPCR analyses of cDNA or immunoprecipitated DNA (see below) were performed using ×2 A-Star Real Time PCR Master Mix (BioFACT) in a Thermo Fisher QuantStudio 5 real-time PCR machine. All qPCR experiments were conducted in three biological replicates, each with three technical replicates.

For RT-qPCR, data analyses were performed according to the previously published ΔΔCT method (Livak and Schmittgen, 2001), with the modification of using two reference genes, *PP2AA3* (*AT1G13320*) and a *SAND* family gene (*AT2G28390*) to normalize the data (Hong et al., 2010). Data normalization was performed using the geometric mean of the two reference genes. Sequences of primers used in RT-qPCR analyses are shown in Supplementary Table 1. The statistical significance of differences in gene expression levels among samples was assessed using one-way ANOVA with a 0.05 level of significance (95% CI).

### Chromatin Immunoprecipitation Assays

Chromatin immunoprecipitation (ChIP) assays were performed using wild-type and *vip3* mutant seedlings, as described previously (Susila et al., 2021). Briefly, seedlings of each genotype were harvested at the 1.02 stage (Boyes et al., 2001) and crosslinked using fixation buffer (1% formaldehyde). Immunoprecipitation was performed with polyclonal anti-H3K4me3 (Millipore, 04-745) or anti-H3K27me3 (Millipore, 07-449) antibodies bound to Dynabead Protein A (Thermo Scientific). The ChIPed DNA was extracted using the ChIP DNA Clean & Concentrator kit (Zymo Research). Relative enrichment of histone modifications was analyzed using qPCR as described earlier (Susila et al., 2021). The primers used in ChIP-qPCR are shown in Supplementary Table 1. All ChIP experiments were performed with three biological replicates and three technical replicates for each genotype.

### Designing and Cloning amiRNAs That Target *MAF* Genes

To posttranscriptionally knock down the *MAF2–MAF5* genes, artificial microRNAs (amiRNAs) were designed using the WMD3 webtool (Schwab et al., 2006). Predominantly expressed splice variants of *MAF2–MAF5* (*MAF2.3*, *MAF3.1*, *MAF4.3*, and *MAF5.2*) were selected from the Araport11 cDNA collection and used as target genes for subsequent studies. Two independent amiRNAs were selected and amplified using *pRS300* as a template with four amiRNA-specific primers (Supplementary Table 1), as previously described (Schwab et al., 2006). The amplified amiRNAs were cloned into the *pENTR2B* vector and subsequently into the *pEG100* vector containing the 35S promoter.

### Protein–Protein Interaction Analyses Using Deep Learning Algorithms

To test whether MAF2–MAF5 interact with SVP, we used two recently developed artificial intelligence (AI)–based deep learning programs, D-SCRIPT (Sledzieski et al., 2021) and PPI-Detect (Romero-Molina et al., 2019). Protein sequences were provided in fasta format as an input for both programs. Protein phosphatase 2A A3 (PP2AA3) was used as a negative interactor control.

### Yeast Two-Hybrid Assays

For confirmation of the physical interaction between MAF2–MAF5 and SVP, the full-length coding sequences of the predominantly expressed splice variants of MAF2–MAF5 (*MAF2.3*, *MAF3.1*, *MAF4.3*, and *MAF5.2*) were fused in-frame to the DNA binding domain (BD) in the *pGBKT7* vector. For the activation domain (AD)–fused SVP, full-length coding sequences of SVP were fused to the GAL4 AD in the *pGADT7* vector. The double transformation was performed by introducing a combination of *SVP* with the *MAF2–MAF5* vectors into the yeast strain AH109.

### Co-immunoprecipitation Assays

Co-immunoprecipitation (Co-IP) experiments were performed in Arabidopsis mesophyll protoplasts as described earlier (Wu et al., 2009). The coding sequences of *MAF2*–*MAF5* were fused with GFP (*35S::MAF-GFP*) in the 326-GFP vector (Lee et al., 2001) and co-transfected with *35S::SVP-2HA* in the protoplasts isolated from wild-type plants. The transfected protoplasts were incubated at 23°C for 3 h to allow production of these proteins in sufficient quantities before overnight incubation at 10°C. After incubation, the protoplasts were pelleted and lysed with lysis buffer (10 mM Tris–HCl, pH 7.5, with .1% Triton X-100, and ×1 Roche Protease Inhibitor Cocktail). The lysate was then incubated overnight with GFP-Trap magnetic beads (Chromotek). Anti-GFP monoclonal antibodies (Roche) and anti-HA high-affinity monoclonal antibody clone 3F10 (Sigma-Aldrich) were used as primary antibodies for the western blots.

## Results

### PAF1C-Deficient Mutants Flower Early at a Broad Range of Temperatures

**Figure 1:**
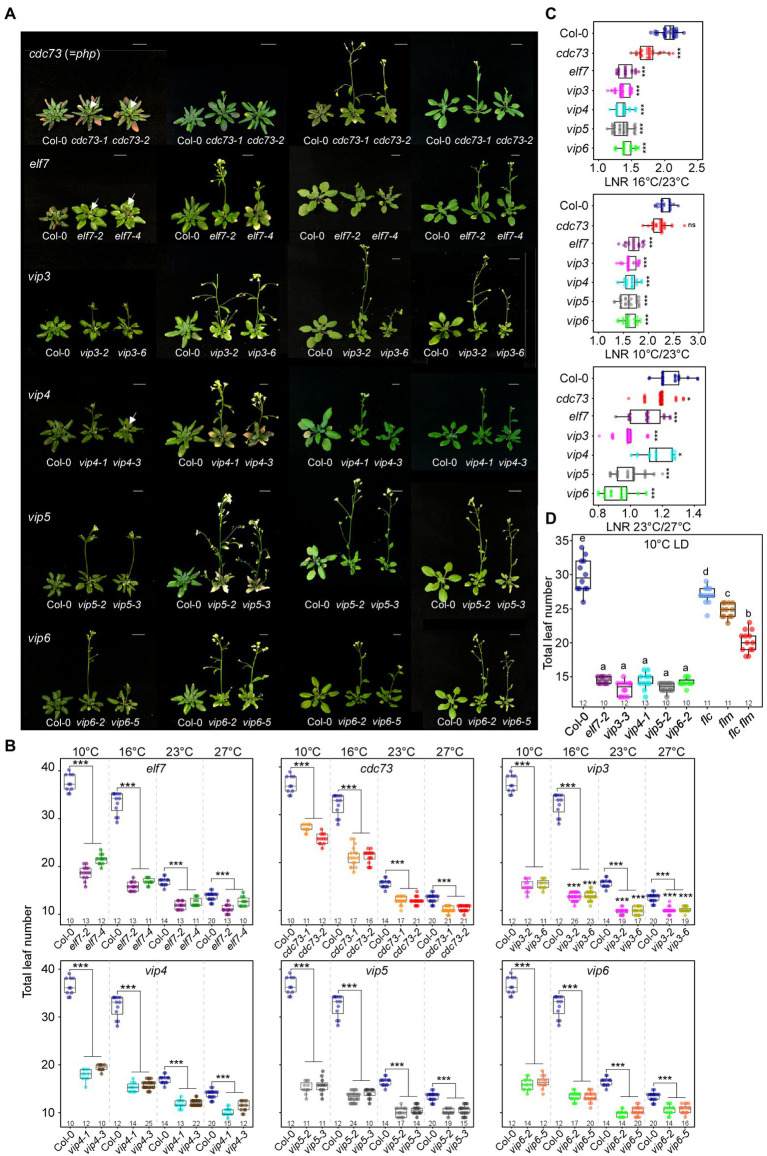
Early flowering of polymerase II–associated factor 1 complex (PAF1C)-deficient mutants at all tested temperatures under long-day (LD) conditions. **(A,B)** Phenotype **(A)** and flowering time quantified as total leaf number **(B)** of PAF1C-deficient mutant lines at 10, 16, 23, and 27°C. Arrows indicate inflorescences. Note that the same leaf number data from Col-0 plants were used in each panel **(B)**. One-way ANOVA followed by Dunnett’s multiple comparison tests were performed to test the statistical significance. Scale bar = 1 cm. **(C)** Leaf number ratio (LNR) of Col-0 and PAF1C-deficient mutants. **(D)** Flowering time comparison of PAF1C-deficient mutants with *flc*, *flm*, and *flc flm* mutants at 10°C. One-way ANOVA followed by Dunnett’s multiple comparison tests were performed to test the statistical significance (*p* < 0.05). ^*^p < 0.05 and ^***^p ≤ 0.001. Letters in **(D)** indicate significant difference by one-way ANOVA followed by Tukey’s range tests Numbers above the *x*-axis represent *n*.

To test the effect of a lesion in PAF1C on flowering time, we measured flowering time of PAF1C-deficient mutants at different temperatures ranging from chilling (i.e., cold but not freezing, 10°C) to high temperature (27°C). To show that any observed flowering time change was not specific to a single allele, we used two independent homozygous mutant lines for each PAF1C gene (*CDC73*, *ELF7*, *VIP3*, *VIP4*, *VIP5*, and *VIP6*; Figures 1A,B). For the previously uncharacterized T-DNA lines *elf7-4* (SALK_070632), *vip5-3* (SALK_055889), and *vip6-5* (SALK_119910), we performed conventional RT-PCR to examine their transcript levels; indeed, all three lines were found to be RNA-null alleles (Supplementary Figure 1).

Flowering time measurement showed that wild-type plants flowered with a mean total leaf number (TLN) of 36.3 ± 1.8, 32.0 ± 2.3, 15.6 ± 0.9, and 12.5 ± 0.5 at 10, 16, 23, and 27°C, respectively (Figures 1A,B; Supplementary Table 2). At all tested temperatures, the PAF1C-deficient mutants flowered significantly earlier than the wild-type plants, indicating that a lesion in PAF1C caused early flowering at a broad range of temperatures. In particular, PAF1C-deficient mutants showed very early flowering, compared with the wild type, as temperature decreased. We selected an allele that showed a strong early flowering phenotype from each gene (*cdc73-2*, *elf7-2*, *vip3-2*, *vip4-1*, *vip5-2*, and *vip6-2*) and used these mutants for further analyses.

To assess the temperature sensitivity of PAF1C-deficient mutants, we calculated the leaf number ratio (LNR) of *cdc73-2*, *elf7-2*, *vip3-2*, *vip4-1*, *vip5-2*, and *vip6-2* mutants, using the TLN values at different temperatures. A LNR close to 1 indicates that temperature has little effect on flowering. All PAF1C-deficient mutants showed significantly lower LNRs compared with wild-type plants (Figure 1C; Supplementary Table 3). These results indicated that a lesion in PAF1C caused ambient temperature-insensitive flowering, especially at lower temperatures.

We then compared flowering time of PAF1C mutants with that of *flc*, *flm*, and *flc flm* mutants at 10°C. The *flc*, *flm*, and *flc flm* mutants flowered with 27.0 ± 1.3, 24.8 ± 1.1, and 20.2 ± 1.6 leaves, respectively, at 10°C (Figure 1D). Interestingly, their flowering times (measured as TLN) were later than *elf7*, *vip3*, *vip4*, *vip5*, and *vip6* single mutants. This indicated that a lesion in both *FLC* and *FLM* was insufficient to phenocopy the early flowering time seen in PAF1C-deficient mutants. Therefore, considering that the PAF1C regulates *FLC* and *FLM* transcription (Zhang and Van Nocker, 2002; Zhang et al., 2003; Oh et al., 2004), these results suggested the possibility that PAF1C regulates other factors, in addition to *FLC* and *FLM*, in modulating flowering time at 10°C.

### Transcriptome Analyses of PAF1C-Deficient Mutants

**Figure 2:**
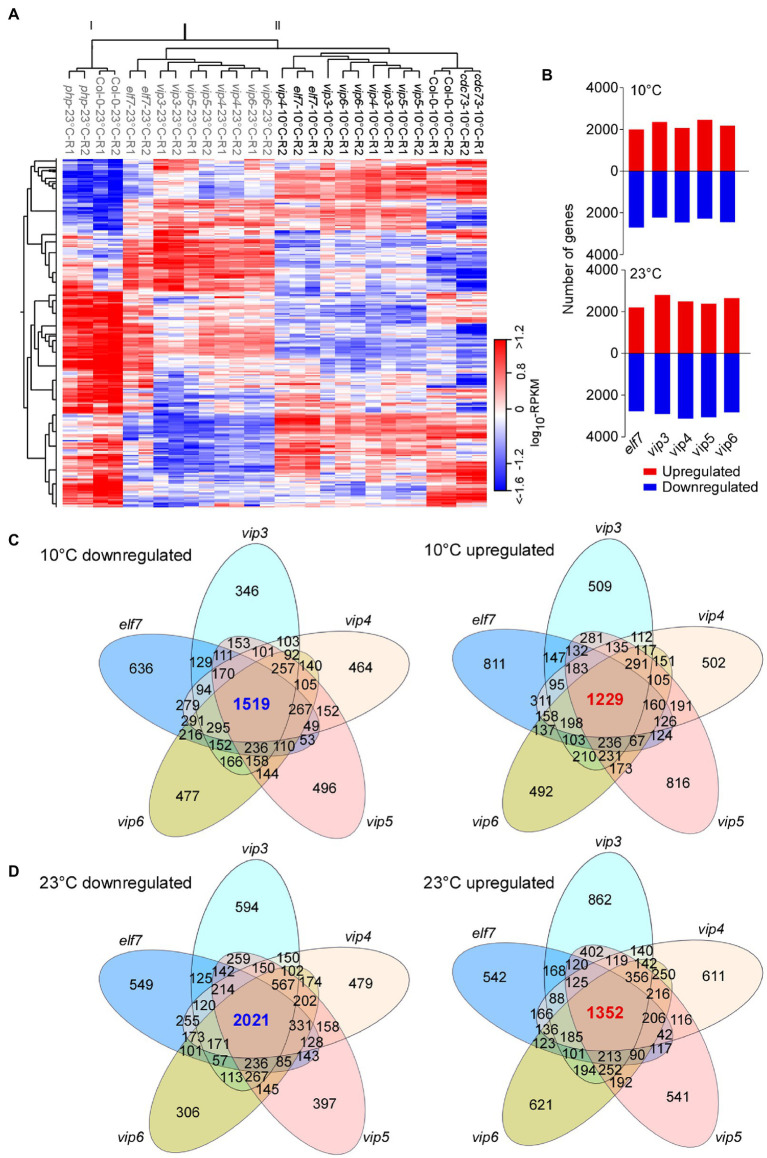
Transcriptome analyses of PAF1C-deficient mutants. **(A)** Heatmap showing the global expression differences between the wild type (Col-0) and PAF1C-deficient mutants. The genotypes of plants grown at 23°C are shown in grey, whereas plants grown at 10°C are shown in black. **(B)** Differentially expressed genes (DEGs) in PAF1C-deficient mutants compared to wild-type (Col-0) seedlings at 10°C (upper panel) and 23°C (lower panel). **(C,D)** Venn diagrams representing the commonly downregulated and upregulated genes at 10°C **(C)** and 23°C **(D)** in PAF1C-deficient mutants.

Mutants in all PAF1C components (except *CDC73*) flowered significantly earlier than *flc flm* double mutants at 10°C (Figure 1D), suggesting that additional factors are involved in this early flowering. To identify these factors, we performed RNA-seq using PAF1C-deficient mutants grown at 10 and 23°C. Euclidean distance clustering associated with complete linkage classified the transcriptome profiles into two major clades (Figure 2A): clade I, containing Col-0 and *cdc73* mutants grown at 23°C; and clade II, containing the remaining mutants grown at 10 and 23°C. This indicates that the Col-0 plants and *cdc73* mutants grown at 23°C have similar transcriptome profiles. Clade II was further divided into plants grown at 23°C and plants grown at 10°C. Among plants grown at 10°C, Col-0 plants and *cdc73* mutants grouped together, whereas *elf7*, *vip3*, *vip4*, *vip5*, and *vip6* mutants grouped together. This expression profile-based classification was consistent with the flowering time changes of PAF1C-deficient mutants at low temperature. As both *cdc73* mutants flowered later than other PAF1C-deficient mutants (Figure 1) and *cdc73* mutants were grouped in the same clade with Col-0 plants based on RNA-seq data (Figure 2A), we excluded *CDC73* from further analyses.

We selected DEGs that showed increased or decreased transcript levels (>1.5-fold). Transcriptome analyses revealed large numbers of DEGs in PAF1C-deficient mutants at both 10 and 23°C (Figure 2B). We then analyzed DEGs that were commonly upregulated and downregulated in different mutants (Figures 2C,D). At 10°C, 1,519 genes were commonly downregulated in *elf7*, *vip3*, *vip4*, *vip5*, and *vip6* mutants, whereas 1,229 genes were commonly upregulated in these mutants (Figure 2C). At 23°C, 2,021 genes were commonly downregulated in *elf7*, *vip3*, *vip4*, *vip5*, and *vip6* mutants, and 1,352 genes were upregulated in these mutants (Figure 2D). These analyses indicated that the largest number of intersecting DEGs was in the set containing the *elf7*, *vip3*, *vip4*, *vip5*, and *vip6* mutants, suggesting that a large number of genes were commonly altered in these mutants at both temperatures. This observation was also consistent with the similar early flowering phenotypes of *elf7*, *vip3*, *vip4*, *vip5*, and *vip6* mutants at 10 and 23°C.

To understand the biological significance of the common DEGs in PAF1C-deficient mutants, we performed Gene Ontology (GO) analyses using the webtool DAVID (Ashburner et al., 2000). At 10°C, the downregulated genes showed significant enrichment for GO terms related to the response to transcription, microtubule-based movement, different metabolic processes, response to jasmonic acid, and stomatal complex development. The upregulated genes were enriched in GO terms related to different metabolic processes and response to different stimuli (Supplementary Figure 2A). At 23°C, the downregulated genes were enriched in GO terms related to response to Karrikin, different metabolic processes, MAPK cascade, and photosynthesis, whereas the upregulated genes were enriched with GO terms related to different metabolic processes, response to oxidative stress, and cell wall organization (Supplementary Figure 2B).

### PAF1C Regulates the Expression of *FLC* and the Other *FLC*-Clade Genes

**Figure 3:**
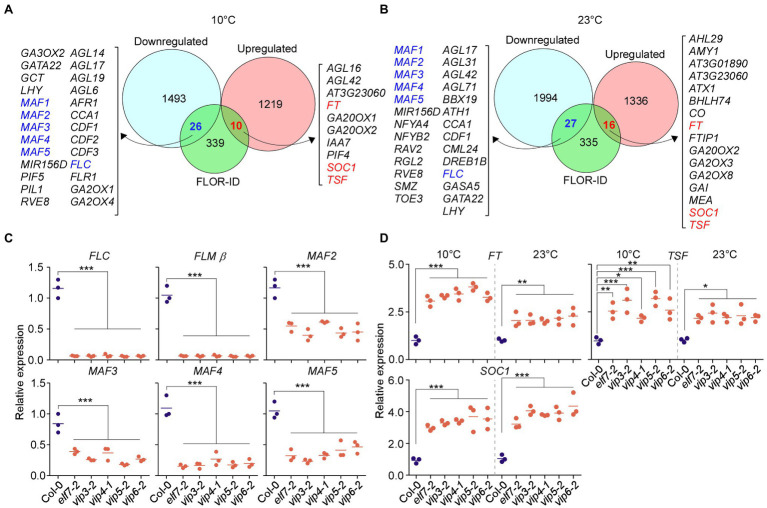
Polymerase II–associated factor 1 complex regulates expression of *FLOWERING LOCUS C* (*FLC*) and the *FLC*-clade genes *MADS AFFECTING FLOWERING1* (*MAF1*)*/FLOWERING LOCUS M* (*FLM*) and *MAF2–MAF5*. **(A,B)** Commonly downregulated and upregulated flowering genes in PAF1C-deficient mutants at 10°C **(A)** and 23°C **(B)**. **(C)** qPCR confirmation of downregulation of *FLC* and *FLC*-clade genes in PAF1C-deficient mutants at 10°C. **(D)** mRNA levels of *FLOWERING LOCUS T* (*FT*), *TSF*, and *SUPPRESSOR OF OVEREXPRESSION OF CO1* (*SOC1*) in PAF1C-deficient mutants at 10 and 23°C. One-way ANOVA followed by Dunnett’s multiple comparison tests were performed to test the statistical significance. ^*^p < 0.05; ^**^p < 0.01; and ^***^p ≤ 0.001.

The Flowering Interactive Database (FLOR-ID; Bouché et al., 2016) contains known genes involved in regulating flowering time. To check whether PAF1C deficiency affects the expression of known flowering time genes, we analyzed which genes in FLOR-ID were included among the common DEGs in PAF1C-deficient mutants at 10 and 23°C (Figures 2C,D). As PAF1C is involved in maintaining the active transcription of its target genes (He et al., 2004; Oh et al., 2004; Yu and Michaels, 2010), we expect that the direct targets of PAF1C will be downregulated in the PAF1C-deficient plants. Interestingly, our analyses showed that 26 and 27 flowering time genes were downregulated at 10 and 23°C, respectively, and both sets included *FLC* and *MAF1*, *2*, *3*, *4*, and *5* (Figures 3A,B).

We performed RT-qPCR analyses to confirm the downregulation of *FLC* and *FLC*-clade genes in PAF1C-deficient mutants at 10°C. Among the alternatively spliced forms produced from the *FLM* locus, *FLM-ß* is the functional form (Scortecci et al., 2001; Pose et al., 2013); therefore, we measured *FLM-ß* transcript levels for *FLM*. The RT-qPCR showed that that *FLC*, *FLM*, and *MAF2*–*MAF5* showed significant downregulation in PAF1C-deficient mutants (Figure 3C). *FLC* showed 17.6-fold downregulation in *elf7-2* mutants, 18-fold in *vip3-2* mutants, 16-fold in *vip4-1* mutants, 20.1-fold in *vip5-2* mutants, and 18.3-fold in *vip6-2* mutants (Figure 3C), consistent with a previous study (Oh et al., 2004). Similarly, *FLM-ß* showed 15.6- to 18.2-fold downregulation in PAF1C-deficient mutants. *MAF2* showed 2.1-fold downregulation in *elf7-2* mutants, 2.9-fold in *vip3-2* mutants, 1.9-fold in *vip4-1* mutants, 2.7-fold in *vip5-2* mutants, and 2.6-fold in *vip6-2* mutants at 10°C. Similarly, *MAF3* was downregulated by 2.1- to 4.6-fold in the PAF1C-deficient mutants at 10°C. *MAF4* mRNA levels were downregulated by 4.1- to 7.1-fold and *MAF5* mRNA levels were downregulated 2.2- to 4.4-fold in the PAF1C-deficient mutants at 10°C.

We then measured the mRNA levels of *FT*, *TSF*, and *SOC1* by RT-qPCR and found that the mRNA levels of *FT*, *TSF*, and *SOC1* were significantly higher in the PAF1C-deficient mutants at 10 and 23°C compared with the wild type. *FT* mRNA levels were increased by 3.1- to 3.8-fold in the PAF1C-deficient mutants at 10°C (Figure 3D). At 23°C, *FT* was upregulated by 1.8- to 2.3-fold. *TSF* was also significantly upregulated in the PAF1C-deficient mutants at both temperatures. *SOC1* transcript levels showed a similar pattern, with a fold increase of 3.2–4.0 and 3.1–4.1 in PAF1C-deficient mutants at 10 and 23°C, respectively, consistent with the downregulation of *FLC* and *FLC*-clade genes in PAF1C-deficient mutants (Figure 3C). These results suggested that functional PAF1C is required for the expression of *FLC* and *FLC*-clade genes and that a lesion in one of its components results in the downregulation of *FLC* and *FLC*-clade genes, which leads to the derepression of *FT*, *TSF*, and *SOC1*, and early flowering. Furthermore, considering that the expression of all *MAF* genes was affected in PAF1C-deficient mutants and PAF1C-deficient mutants flowered significantly earlier than *flc flm* double mutants (Figure 1D), it is likely that the *MAF2–MAF5* genes play a significant role in floral repression at chilling temperatures. Although *FLC* and *FLC*-clade genes are known to undergo alternative splicing and their splice variants might have differential effects on flowering time (Caicedo et al., 2004; Lee et al., 2013; Pose et al., 2013; Rosloski et al., 2013), our RNA-seq data showed no significant difference in the levels of the splice variants of *FLC* and *FLC*-clade genes (Supplementary Figure 3), except for their overall downregulation, suggesting that the flowering time change seen in PAF1C-decificient mutants was not associated with the differential splicing patterns of *FLC* and *FLC*-clade genes.

### PAF1C Deficiency Alters the Epigenetic Status of the *FLC*-Clade Genes

**Figure 4:**
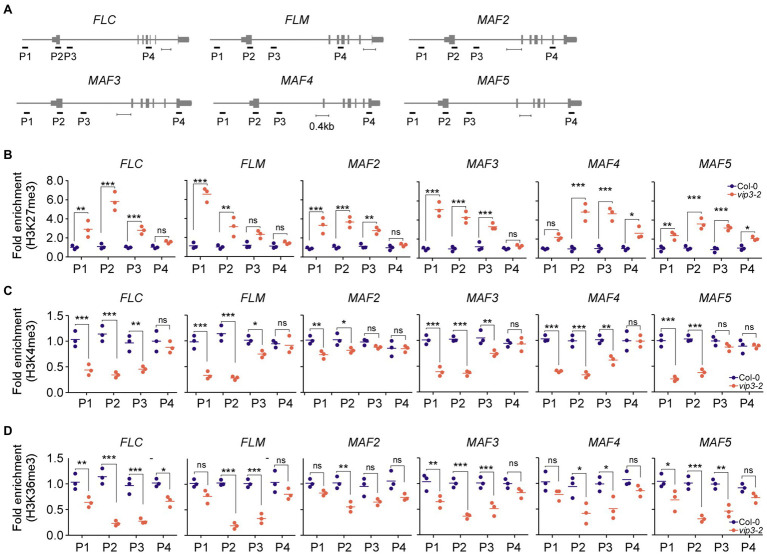
Polymerase II–associated factor 1 complex deficiency alters the epigenetic status of chromatin of the *FLC* and the *FLC*-clade genes *MAF1/FLM* and *MAF2–MAF5*. **(A)** Schematic representation of the genomic regions of *FLC* and *FLC*-clade genes and the regions amplified for chromatin immunoprecipitation (ChIP) assays. Scale bar = 0.4 kb. Note that our P2 region in *FLC* partially overlaps with the region that was used in a previous study (He et al., 2004). In the case of *FLM*, our P2 region is included in a region that was previously shown to be affected (He et al., 2004). **(B–D)** Fold enrichment of the repressive epigenetic H3K27me3 marks **(B)**, permissive H3K4me3 marks **(C)**, and permissive H3K36me3 marks **(D)** at the genomic regions of *FLC* and *FLC*-clade genes in wild-type (Col-0) and PAF1C-deficient *vip3* mutant seedlings. For each primer pair, the enrichment was normalized to the wild-type control (Col-0). One-way ANOVA followed by Dunnett’s multiple comparison tests were performed to test the statistical significance. ^*^p < 0.05; ^**^p < 0.01; ^***^p ≤ 0.001; and ns: not significant.

Our expression analyses showed that in addition to *FLC* and *FLM*, *MAF2–MAF5* were downregulated in PAF1C-deficient mutants (Figure 3), suggesting that this downregulation may be due to the altered epigenetic status at these loci in PAF1C-deficient mutants. To test this possibility, we analyzed the levels of the repressive H3K27me3 marks and permissive H3K4me3/H3K36me3 marks of *FLC* and the *FLC*-clade genes in *vip3-2* mutants (as a representative PAF1C-deficient mutant line) and wild-type plants grown at 10°C. Four different qPCR primer sets (P1–P4), spanning the entire gene bodies of the target genes, were used to assess the enrichment of the repressive/permissive marks (Figure 4A).

Consistent with their downregulation, the *FLC* and *FLC*-clade genes showed significantly increased enrichment of the repressive H3K27me3 marks throughout their gene bodies in *vip3* mutants (Figure 4B). In *FLC*, the highest enrichment (5.8-fold higher enrichment compared to wild-type plants) of repressive H3K27me3 marks was observed in the P2 region, which contains the transcription start site (Figure 4B), consistent with a previous finding (He et al., 2004). Significantly higher enrichment was also observed in the P1 (2.9-fold) and P3 (2.8-fold) regions of *FLC*. In *FLM* chromatin, the H3K27me3 enrichment was highest in the P1 region (5.8-fold) followed by P2 (3.1-fold) and P3 (2.0-fold) regions. *MAF2* and *MAF3* showed similar H3K27me3 patterns with the highest enrichment in the P1 region (3.7- and 5.3-fold, respectively), followed by P2 (3.6- and 4.4-fold, respectively) and P3 regions (2.4- and 2.7-fold, respectively). The enrichment of H3K27me3 in the *MAF5* chromatin was highest in the P3 and P2 regions (3.5- and 3.4-fold, respectively) followed by P1 with 2.4-fold higher enrichment of H3K27me3 in *vip3* mutants compared to wild-type plants at 10°C. H3K27me3 enrichment in the P4 regions of *FLC*, *FLM*, *MAF2*, and *MAF3* of *vip3* mutants was comparable with wild-type samples, whereas *MAF4* and *MAF5* showed slightly higher enrichment in the P4 regions (1.3- and 2.5-fold, respectively) in *vip3* mutants (Figure 4B).

By contrast, enrichment of the permissive H3K4me3 marks was significantly reduced in the chromatin of *FLC* and *FLC*-clade genes in *vip3* mutants, primarily in the P1 and P2 regions (Figure 4C). Enrichment of H3K4me3 in the *FLC* chromatin in *vip3* mutants was reduced 3.3-fold in the P2 region, 2.4-fold in P1, and 2.1-fold in P3 compared to wild-type plants. *FLM* also showed reduced enrichment of H3K4me3 marks in the P2 (4.1-fold) and P1 (2.9-fold) regions. In the *MAF2* chromatin, reduced H3K4me3 enrichment was seen in the P1 (1.4-fold) and P2 (1.3-fold) regions. In addition, in the *MAF3*-*5* chromatin, H3K4me3 enrichment was significantly lower in the P2 region (2.8-, 3.0-, and 2.7-fold, respectively) and the P1 region (2.6-, 2.5-, and 3.9-fold, respectively) in *vip3* mutants (Figure 4C). Similar reduction patterns were found for the H3K36me3 mark at the gene bodies of these genes, with significantly reduced enrichment at the P2 and P3 regions (Figure 4D). Taken together, these results suggest that PAF1C is required to maintain permissive epigenetic marks and prevent deposition of repressive marks at the *FLC* and *FLC*-clade genes, thereby maintaining their active transcription.

### *FLC*-Clade Genes Are Upregulated in Wild-Type Plants at 10°C

**Figure 5:**
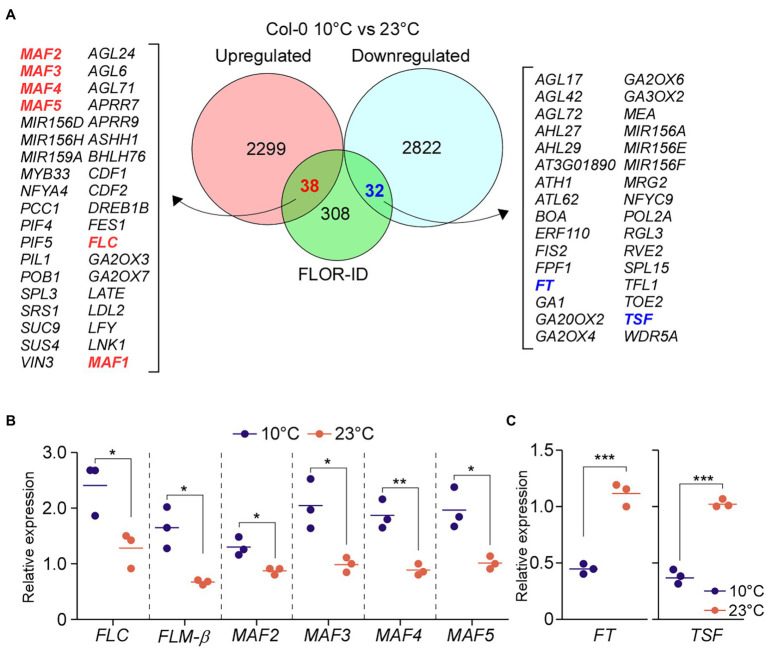
Low temperature induces the expression of *FLC* and *FLC*-clade genes. **(A)** Differentially expressed genes (DEGs) between wild-type (Col-0) seedlings grown at 10 and 23°C under LD conditions. **(B,C)** Relative mRNA levels of *FLC* and *FLC*-clade genes **(B)** and *FT* **(C)** at 10°C under LD conditions. One-way ANOVA followed by Dunnett’s multiple comparison tests were performed to test the statistical significance. ^*^p < 0.05; ^**^p < 0.01; and ^***^p ≤ 0.001.

The early flowering of PAF1C-deficient mutants at 10°C is likely due to the combinatorial effect of FLC, FLM, and the other MAFs, suggesting the functional importance of MAF2–MAF5 at low temperature (10°C). To test whether expression of these genes is upregulated in wild-type plants at 10°C, we compared the transcript levels of these genes in wild-type plants at 10 and 23°C using our RNA-seq data. This analysis revealed upregulation of *FLC*, *FLM*, and *MAF2–MAF5* in wild-type plants at 10°C compared to 23°C by at least 1.5-fold (Figure 5A). RT-qPCR analyses also showed statistically significant induction of *FLC*, *FLM*, and *MAF2*–*MAF5* in wild-type plants at 10°C compared to 23°C (Figure 5B). Consistent with the upregulation of *FLC* and *FLC*-clade genes, transcript levels of *FT* and *TSF*, their downstream targets, were significantly reduced (>3-fold) in wild-type plants at 10°C in comparison to 23°C (Figure 5C). This suggests that these MAFs might play important roles in modulating flowering time at chilling temperatures by regulating *FT* and *TSF*.

### *FLC*-Clade Genes Are Important for Floral Repression at Chilling Temperatures

**Figure 6:**
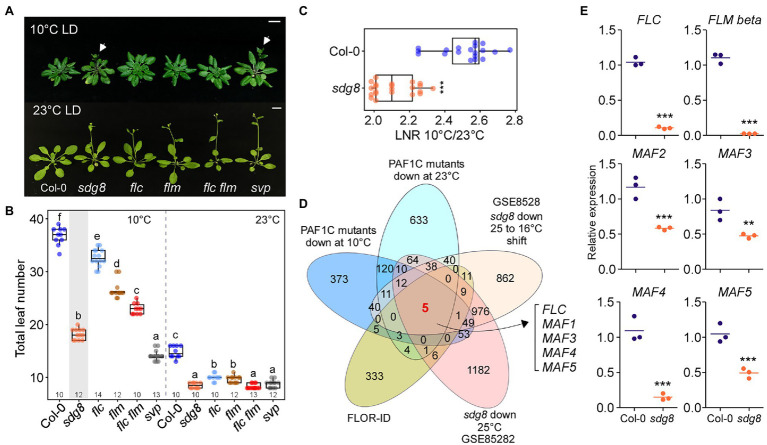
*MAF* genes are important for floral repression at low temperature. **(A,B)** Flowering phenotype **(A)** and flowering time **(B)** of *sdg8*, *flc*, *flm*, *flc flm*, and *short vegetative phase* (*svp*) mutants at 10 and 23°C under LD conditions. Arrows indicate inflorescences. **(C)** Leaf number ratio (LNR; 10°C/23°C) of *sdg8* mutants. **(D)** Venn diagram of the genes that were commonly downregulated in *sdg8* mutants and PAF1C-deficient mutants at different temperatures. **(E)** Reverse transcription quantitative PCR (RT-qPCR) of *FLC* and *FLC*-clade genes in the wild type (Col-0) and *sdg8* mutants at 10°C under LD conditions. One-way ANOVA followed by Dunnett’s multiple comparison tests were performed to test the statistical significance. ^*^p < 0.05; ^**^p < 0.01; ^***^p ≤ 0.001; and ns: not significant. Letters indicate significant differences by one-way ANOVA followed by Tukey’s range tests (*p* < 0.05). Numbers above the *x*-axis in **(B)** represent *n*. Scale bar = 1 cm.

Polymerase II–associated factor 1 complex modulates the expression of downstream genes by recruiting a number of histone modifiers, including SET DOMAIN GROUP8 (SDG8; Wood et al., 2003; Ng et al., 2003a; Kim et al., 2005). SDG8 recruited by PAF1C then regulates the expression of *FLC* and *FLM*; therefore, a mutation in *SDG8* causes early flowering at normal temperatures (Kim et al., 2005). Because the flowering response of *sdg8* mutants under chilling-stress temperatures is not known, we analyzed the flowering time of *sdg8* mutants at 10 and 23°C. We found that the *sdg8* mutants flowered significantly earlier than *flc*, *flm*, and *flc flm* mutants at 10°C (Figures 6A,B). The *sdg8* mutants flowered with 18.1 ± 1.0 leaves, which was significantly earlier than *flc* mutants (32.6 ± 1.6 leaves), *flm* mutants (26.5 ± 1.7 leaves), and *flc flm* double mutants (23.0 ± 1.1 leaves) at 10°C. However, at 23°C, the flowering time of *sdg8* mutants (8.5 ± .6 leaves) was only slightly earlier than *flc* and *flm* mutants (10.0 ± 0.7 and 9.7 ± 0.7 leaves, respectively) and was comparable to *flc flm* double mutants (8.4 ± .5 leaves; Figure 6B). LNR analyses revealed significantly decreased LNR values of *sdg8* mutants to low temperature (Figure 6C), indicating that the temperature responsiveness of *sdg8* mutants was reduced.

To determine whether *MAF* genes play a role in the regulation of flowering time in *sdg8* mutants, we first analyzed publicly available RNA-seq data for *sdg8* mutants grown at 16°C with or without shifting to 25°C (GSE85282; Pajoro et al., 2017) and then found the intersection of (1) the set of DEGs in *sdg8* mutants, (2) the genes that were commonly downregulated in PAF1C-deficient mutants at 10°C (Figure 2C) and 23°C (Figure 2D), and (3) the list of flowering time genes from FLOR-ID (Bouché et al., 2016). From this comparison, we found that *FLC*, *FLM*, and *MAF3*–*MAF5* were commonly downregulated in *sdg8* mutants (Figure 6D; Supplementary Figure 4), like in PAF1C-deficient mutants (Figure 3). *MAF2*, which showed 1.8-fold downregulation in *sdg8* mutants, was not identified here, due to the criteria for selecting DEGs (2-fold change).

We then performed RT-qPCR to confirm the downregulation of these genes at 10°C. The RT-qPCR results showed statistically significant downregulation of *FLC*, *FLM*-*ß*, and *MAF2–MAF5* mRNA levels in *sdg8* mutants at 10°C (Figure 6E). *FLC* mRNA levels were decreased by 13.2-fold, whereas *FLM-ß* showed 15.9-fold downregulation in *sdg8* mutants at 10°C. In addition, expression of *MAF2–MAF5* was downregulated by 2.0-, 1.7-, 7.3-, and 1.9-fold, respectively, in *sdg8* mutants at 10°C (Figure 6E). Taken together, these results suggested that the early flowering phenotype of *sdg8* mutants at chilling temperature is mediated by the downregulation of *FLC* and *MAF* genes. However, it should be noted that *sdg8* mutants flowered slightly later than PAF1C-dificient mutants at 10°C (Supplementary Figure 5), suggesting a possibility that the PAF1C recruits an additional histone modifier(s), besides SDG8, to regulate the expression of the *FLC*-clade genes.

### Knockout/Knockdown of *FLC* and *FLC*-Clade Genes Results in Extremely Early Flowering at 10°C

**Figure 7:**
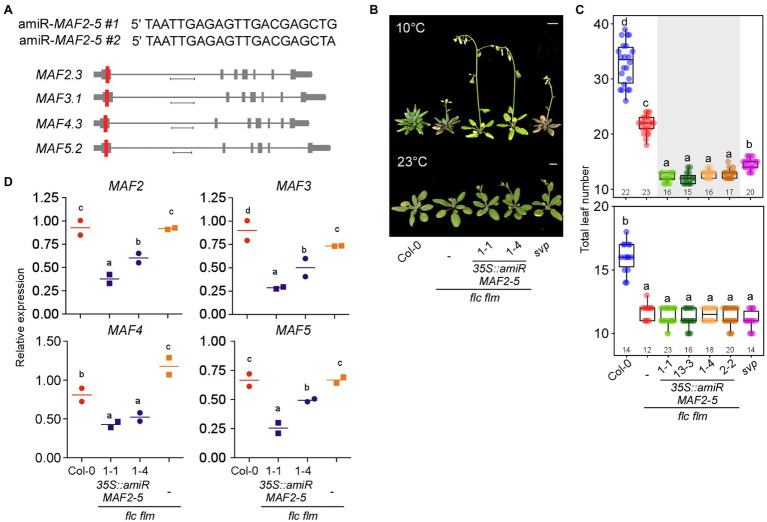
Knockout/down of *FLC* and *MAFs* together results in precocious flowering at chilling temperatures. **(A)** Sequences of artificial microRNAs (amiRNAs) designed to knock down *MAF2–MAF5* genes (top) and schematic representation of their location (red vertical line) in a predominantly expressed spliced variant of *MAF2–MAF5* genes (bottom). Scale bar: 0.4 kb **(B,C)** Flowering phenotype **(B)** and flowering time **(C)** of *35S::amiR-MAF2-5 flc flm* transgenic seedlings at 10 and 23°C under LD conditions. Scale bar = 1 cm. **(D)** RT-qPCR confirmation of knockdown of *MAF2–MAF5* in *35S::amiR-MAF2-5 flc flm* transgenic lines. Letters indicate significant differences by one-way ANOVA followed by Tukey’s range tests (*p* < 0.05). Numbers above the *x*-axis in **(C)** represent *n*.

To genetically confirm the importance of *MAF* genes in repressing flowering at chilling temperatures, we used amiRNAs to repress the *MAF* genes. To this end, we designed two amiRNAs that simultaneously target *MAF2*–*MAF5* (Figure 7A) and overexpressed these amiRNAs in *flc flm* double mutants. Two independent transgenic lines overexpressing amiRNAs against *MAF2*–*MAF5* (*35S::amiR-MAF2-5 flc flm #1* and *#2*) flowered significantly earlier than *flc flm* double mutants (Figures 7B,C). At 10°C, The *35S::amiR-MAF2-5 flc flm #1* and *#2* plants flowered with an average TLN of 12.1 to 12.9 leaves (c.f., wild-type plants: 32.8 ± 3.8), indicating that knockout/down of *FLC* and all five *FLC*-clade genes caused extremely early flowering at 10°C. Furthermore, these transgenic lines flowered earlier than *flc flm* double mutants (21.6 ± 1.6 leaves; Figure 7C). Interestingly, both *35S::amiR-MAF2-5 flc flm* lines flowered earlier than *svp* mutants (14.6 ± 1.4 leaves). However, at 23°C, The *35S::amiR-MAF2-5 flc flm #1* and *#2* plants flowered with an average TLN of 11.1–11.4 leaves (c.f., wild-type plants: 16.0 ± 1.1), which was similar to the TLN of *flc flm* double (11.7 ± 0.6 leaves) and *svp* single mutants (11.1 ± 0.7 leaves; Figure 7C). These results highlighted the importance of the MAF2–MAF5 transcription factors in repressing flowering at chilling temperatures.

To confirm that this early flowering of *35S::amiR-MAF2-5 flc flm #1* (1–1 and 13–3) and *#2* (1–4 and 2–2) plants was indeed due to the downregulation of *MAF2*–*MAF5*, we performed RT-qPCR analyses. These analyses confirmed the transgenic seedlings showed significantly lower *MAF* mRNA levels compared with the wild-type plants and *flc flm* double mutants (Figure 7D). Furthermore, *MAF2*–*MAF5* mRNA levels were significantly lower in *35S::amiR-MAF2-5 flc flm #1* plants than in *35S::amiR-MAF2-5 flc flm #2* plants, except *MAF4*. The stronger reduction of *MAF* transcript levels in the *35S::amiR-MAF2-5 flc flm #1* plants was consistent with their earlier flowering time phenotype compared with *35S::amiR-MAF2-5 flc flm #2* plants (Figure 7C). Taken together, these data suggest that ablation of function of *FLC* and all *FLC*-clade members resulted in earlier flowering than *flc flm* mutants at chilling temperatures; therefore, MAF2–MAF5 also play a role in repressing flowering at chilling temperatures.

### MAFs Physically Interact With SVP to Form Repressor Complexes

Loss of *SVP* function results in early flowering across a broad range of temperatures (10°C–27°C; Lee et al., 2013). SVP interacts with FLC (Fujiwara et al., 2008; Li et al., 2008) and with FLM (Lee et al., 2013; Pose et al., 2013). *In vivo* and yeast two-hybrid (Y2H) analyses showed that SVP interacts with MAF2 and MAF4 (Gu et al., 2013). Since the *35S::amiR-MAF2-5 flc flm* (*#1* and *#2*) plants showed significantly earlier flowering at 10°C compared with *svp* mutants (Figure 7C), one possible scenario is that SVP alone is insufficient to repress flowering at 10°C and may require FLC-clade proteins to repress flowering.

**Figure 8:**
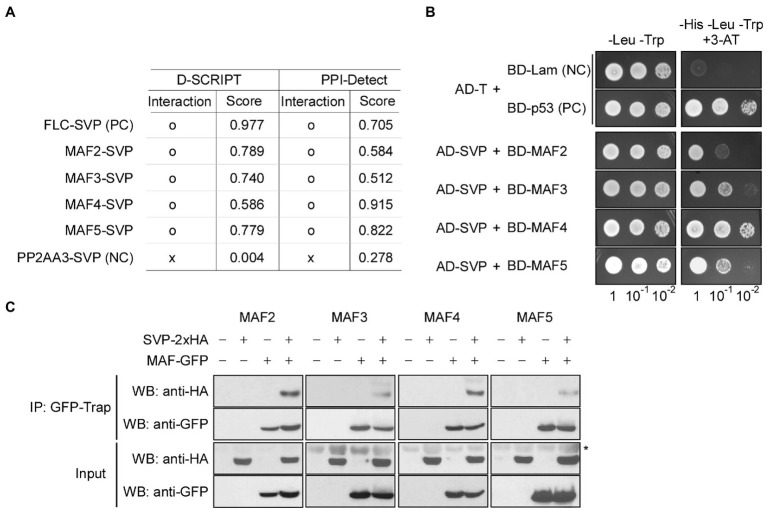
MAFs physically interact with SVP. **(A)** Artificial intelligence (AI)–based prediction of interaction between MAFs and SVP. o: interaction, x: no interaction. Score (ranging from 0 to 1) represents the likelihood of interaction between two input proteins, where 0 represents no predicted interaction and 1 represents a high-confidence prediction that the proteins interact. **(B)** Yeast two-hybrid assays showing the interaction between MAFs and SVP. Positive control (PC): AD-T (SV40 large T-antigen) and BD-p53 (murine p53); negative control (NC): AD-T and BD-Lam (Lamin). **(C)** *In vivo* confirmation of MAF–SVP interactions *via* co-immunoprecipitation assays using Arabidopsis protoplasts at 10°C. PC; positive control, NC; negative control. The asterisk (^*^) represents a nonspecific signal on the anti-HA blot.

To test whether FLC-clade proteins physically interact with SVP, we first used two recently developed artificial intelligence (AI)–based deep learning programs that were designed to predict protein–protein interactions: D-SCRIPT (Sledzieski et al., 2021) and PPI-Detect (Romero-Molina et al., 2019). These programs produce an interaction score between 0 (no interaction predicted) and 1 (interaction strongly predicted). In these analyses, FLC was used as a known interacting partner of SVP (Li et al., 2008), and PP2AA3 was used a negative control. The FLC–SVP interaction scores were 0.977 (D-SCRIPT) and 0.705 (PPI-Detect), whereas the PP2AA3–SVP interaction scores were 0.004 (D-SCRIPT) and 0.278 (PPI-Detect; Figure 8A). From D-SCRIPT analyses, the MAF2–SVP, MAF3–SVP, MAF4–SVP, and MAF5–SVP interaction scores were 0.789, 0.740, 0.586, and 0.779, respectively. From PPI-Detect analyses, the MAF2–SVP, MAF3–SVP, MAF4–SVP, and MAF5–SVP interaction scores were 0.584, 0.512, 0.915, and 0.822, respectively. All of these interaction scores were above the cut-off value of 0.5, suggesting that SVP interacts with MAF2–MAF5 *in vivo*.

We then performed Y2H analyses to experimentally validate the predicted interactions. Indeed, Y2H analyses showed that SVP interacts with MAF2–MAF5 in yeast cells (Figure 8B). To further test these interactions *in vivo*, we performed Co-IP experiments using Arabidopsis mesophyll protoplasts. To this end, *35S::2 × HA:SVP* and *35S::GFP:MAF* vectors were transiently co-expressed in protoplasts to produce HA-tagged SVP and GFP-tagged MAF2–MAF5 proteins and then the transfected protoplasts were shifted to 10°C to test the protein–protein interaction at 10°C. We precipitated protein extracts using GFP-Trap and probed the resulting precipitates with anti-HA antibodies. SVP-2 × HA successfully co-immunoprecipitated with each MAF transcription factor (Figure 8C; asterisk), confirming the interactions between SVP and MAFs. Taken together, these results suggest that MAF2–MAF5, like FLC and FLM, physically interact with SVP, further implying that SVP forms a repressor complex including FLC and MAFs. It is therefore likely that the formation of the complex leads to efficient floral repression, thus allowing the plant to acclimate to chilling temperatures.

### SVP-Mediated Floral Repression Likely Requires *FLC* and *FLC*-Clade Genes

**Figure 9:**
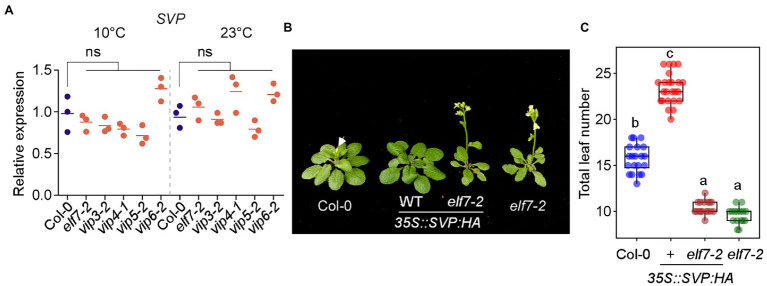
SVP-mediated floral repression is dependent on *FLC* and *FLC*-clade genes. **(A)** *SVP* mRNA levels in PAF1C-deficient mutants at 10 and 23°C under LD conditions. ns; not significant. **(B,C)** Phenotype **(B)** and total leaf number **(C)** of *35S::SVP-HA* and *35S::SVP-HA elf7* plants. Note that these plants were first grown on medium containing phosphinothricin for 6 days at 23°C, transferred to soil, and then grown at 16°C. Letters indicate significant differences by one-way ANOVA followed by Tukey’s range tests (*p* < 0.05).

Since the PAF1C-deficient mutants showed strong early flowering at 10°C (Figure 1), we tested whether *SVP* transcript levels were affected in PAF1C-deficient mutants, as *svp* mutants flower early across a range of temperatures (10°C–27°C; Lee et al., 2013). RT-qPCR analyses showed that *SVP* mRNA levels in PAF1C-deficient mutants were similar to those of Col-0 plants at both 10 and 23°C (Figure 9A; He et al., 2004). This suggested that the flowering time change seen in PAF1C-deficient mutants at both 10 and 23°C was independent of *SVP* transcript levels.

We then tested whether SVP requires PAF1C to delay flowering time. For this experiment, we used *elf7-2* and *vip4-1* mutants as representative PAF1C-deficient mutants and crossed them with *35S::SVP:HA* plants. The *35S::SVP:HA* plants showed delayed flowering (23.3 ± 1.7 leaves) at 23°C, but the *35S::SVP:HA elf7-2* plants flowered with 10.4 ± .7 leaves (Figures 9B,C). Considering that *elf7-2* mutants flowered with 9.6 ± 0.9 leaves at 23°C, this genetic interaction study showed that the late flowering caused by *SVP* overexpression was almost completely suppressed by *elf7-2* mutation. Similarly, the late flowering of *35S::SVP:HA* plants was strongly suppressed by *vip4-1* mutation (Supplementary Figure 6). These results suggested that *SVP* overexpression was unable to delay flowering in the absence of a functional PAF1C.

Short vegetative phase was unable to repress flowering in *elf7-2* mutants, which have dramatically decreased mRNA levels of *FLC* and *FLC*-clade genes (Figure 3B), suggesting the possibility that SVP binding to its targets requires functional FLC and FLC-clade transcription factors. To test this hypothesis, we took advantage of a publicly available ChIP-seq dataset (GSE54881) for SVP-GFP in the presence/absence of *FLC* (*FRI FLC* and *FRI flc*; Mateos et al., 2015). Consistent with a previous study (Mateos et al., 2015), the number of targets bound by SVP-GFP was substantially reduced in plants without functional FLC (*FRI flc*), compared to the plants with functional FLC (*FRI FLC*; Supplementary Figure 7). In terms of the number of bound targets, SVP-GFP was only able to bind to 39.2% of its target genes in the absence of functional FLC (the number of targets bound by SVP-GFP in *FRI FLC* was set to 100%; Supplementary Figure 7A). Furthermore, SVP-GFP was able to bind to 553 additional target genes in the presence of FLC (Supplementary Figure 7B; Mateos et al., 2015), indicating the importance of FLC for SVP binding ability. Since other FLC-clade transcription factors also interact with SVP (Figure 8), these results suggest that the FLC-clade transcription factors play a similar role, especially at low temperatures.

## Discussion

In Arabidopsis, PAF1C regulates flowering primarily through epigenetic modulation of *FLC* and *FLM* expression under standard growth conditions (Kim et al., 2005; Yu and Michaels, 2010). However, the role of PAF1C in regulating flowering time at chilling temperatures remains unknown. In this study, we show that PAF1C not only regulates *FLC* and *FLM*, but also regulates the entire *FLC* clade of genes (*FLM/MAF1* and *MAF2–MAF5*), which play important roles in repressing flowering at low temperatures.

Several environmental factors, including temperature, affect flowering. At lower temperatures, Arabidopsis plants flower late compared to plants at elevated temperatures (Lee et al., 2013). Several MADS-box transcription factors, including FLC, FLM, and SVP, play an important role in delaying flowering (Lee et al., 2007, 2013). *FLC* and *FLM* are well-known to be epigenetically regulated by a number of histone modifiers, including SET domain-containing histone methyltransferases (He et al., 2003; Zhang et al., 2003; Oh et al., 2004; Nasim et al., 2021). PAF1C recruits these histone modifiers to modulate expression of its target genes, including *FLC*, *FLM*, and *MAF2* (Zhang and Van Nocker, 2002; He et al., 2003; Zhang et al., 2003; Oh et al., 2004).

Polymerase II–associated factor 1 complex function is critical for proper plant growth and development, as lesions in PAF1C components result in strong defects in the vegetative and reproductive stages, such as severely stunted growth, stem cell maintenance defects, floral structure defects, and male sterility (Zhang et al., 2003; He et al., 2004; Oh et al., 2004; Fal et al., 2019). Flowering time analyses across a range of ambient temperatures showed that all PAF1C mutants flowered early in a temperature-independent manner with reduced expression of *FLC* and *FLC*-clade genes (Figures 1, 3). This observation validates the previous findings that mutations in *VIP3*, *VIP5*, and *VIP6* result in reduced *FLC* and *FLM* expression and, hence, accelerated flowering (Zhang et al., 2003; Oh et al., 2004). However, flowering of *flc*, *flm*, and *flc flm* mutants was delayed at a chilling temperature of 10°C, indicating that these mutants showed temperature-sensitive flowering at 10°C. This further indicated that *FLC* and *FLM* function primarily from 16 to 27°C (Lee et al., 2013), suggesting that other genes may play important roles at lower temperatures. In this study, we observed that PAF1C-deficient mutants had lower transcript levels of *FLC* and the other *FLC*-clade genes at 10°C (Figure 3), indicating that *FLC*-clade genes are involved in repressing flowering at 10°C. Our ChIP-qPCR assays showed that downregulation of *FLC* and *FLC*-clade genes in PAF1C-deficient mutants is associated with higher enrichment of the repressive mark H3K27me3 and reduced levels of the permissive marks H3K4me3 and H3K36me3 in the chromatin of these genes (Figure 4). This is consistent with previous findings that PAF1C-deficient mutants had reduced *FLC* and *FLM* expression due to the reduced H3K4me3 levels at these loci (He et al., 2004; Oh et al., 2004; Xu et al., 2008). Moreover, we showed that PAF1C-mediated epigenetic regulation is not limited to *FLC* and *FLM*, but also affects the entire *FLC* clade.

A previous study showed that *MAF3* function is more important at lower temperatures than higher temperatures, as the *flc flm maf3* triple mutants flowered earlier than *flc flm* double mutants at 16°C, compared to 23°C (Gu et al., 2013). This supports our hypothesis that *FLC*-clade genes are important to repress flowering at low temperatures, as the *35S::amiR-MAF2-5 flc flm* plants flowered significantly earlier than the *flc flm* double mutants. Furthermore, we observed induction of *FLC* and *FLC*-clade genes in wild-type plants at 10°C (Figure 5), supporting previous findings that the mRNA levels of *FLC* and *FLM-ß* increased at low temperatures (16°C compared to 23°C; Lee et al., 2007; Pose et al., 2013). This might mean that plants increase transcription of these *FLC* family genes in response to low temperatures to ensure efficient floral repression, and this increase in transcription likely requires PAF1C. Since PAF1C components and functions are conserved from unicellular yeast to complex eukaryotic organisms (Tomson and Arndt, 2013), this regulatory mechanism might be conserved and have important functions in other plant species.

One important question raised by our observations is how these MAFs play such a critical role in floral repression at 10°C. One possible answer is their effect on SVP function. MADS-box transcription factors physically interact to form larger complexes that synergistically enhance their abilities to regulate transcription. Consistent with this, the MADS-box transcription factors FLC (Fujiwara et al., 2008; Li et al., 2008) and FLM (Lee et al., 2013) form floral repressor complexes with SVP and enhance their repression of flowering. Our data revealed that SVP interacts with all five MAF transcription factors *in vitro* and *in vivo* (Figure 8). This finding is consistent with our observation that in plants that lack (or have downregulated expression of) *FLC* and *FLC*-clade transcription factor genes, such as the PAF1C-deficient mutants and *35S::amiR-MAF2-5 flc flm* plants, SVP alone is not sufficient to repress flowering, especially at lower temperatures. Consistent with this notion, SVP overexpression was unable to delay flowering in PAF1C-deficient *elf7* and *vip4* mutants (Figure 9; Supplementary Figure 6), with significantly low levels of *FLC* and *FLC*-clade transcripts, suggesting that SVP function depends on FLC and FLC-clade transcription factors, and thus providing novel insight into SVP protein function at low temperatures. However, it should be noted that further genetic interaction analyses, such as analyses of plants overexpressing *SVP* in the *35S::amiR-MAF2-5 flc flm* background, will provide more direct genetic evidence.

Furthermore, our analysis of a previously published ChIP-seq dataset suggested that the presence/absence of FLC influences SVP binding to its targets, as the number of SVP-bound targets nearly doubled in the presence of functional FLC, implying that SVP function depends on FLC, as previously reported (Mateos et al., 2015). It is likely that FLC-clade transcription factors play similar roles, enhancing SVP binding to its targets and/or enhancing its ability to repress transcription; however, further experiments are required to confirm this hypothesis. It would be interesting to perform a genome-wide analysis of whether SVP can bind and repress its target genes in plants with reduced expression of *FLC* and *FLC*-clade genes, such as PAF1-deficient mutants or the *35S::amiR-MAF2-5 flc flm* plants.

In conclusion, our findings showed that PAF1C epigenetically regulates all the *FLC*-clade genes and that these genes play an important role in repressing flowering at chilling temperatures by forming floral repressor complexes with SVP. Wild-type plants accumulate higher levels of *FLC*-clade transcripts in response to chilling temperature to prevent precocious flowering. Our work uncovers the functional importance of MAF transcription factors in repressing flowering at chilling temperatures and increases the current understanding of how flowering is regulated in response to temperature.

## Data Availability Statement

The datasets presented in this study can be found in online repositories. The names of the repository/repositories and accession number(s) can be found at: https://www.ncbi.nlm.nih.gov/geo/, under the accession number GSE171778.

## Author Contributions

ZN and JA designed the research. ZN performed the bioinformatic analyses and conducted experimental work. HS helped with ChIP-qPCR experiments. SJ and GY provided technical assistance. JA supervised the study. All authors contributed to the article and approved the submitted version.

## Funding

This work was supported by a National Research Foundation (NRF) of Korea grant funded by the Korean government (NRF-2017R1A2B3009624 to JA) and Samsung Science and Technology Foundation under Project Number SSTF-BA1602-12.

## Conflict of Interest

The authors declare that the research was conducted in the absence of any commercial or financial relationships that could be construed as a potential conflict of interest.

## Publisher’s Note

All claims expressed in this article are solely those of the authors and do not necessarily represent those of their affiliated organizations, or those of the publisher, the editors and the reviewers. Any product that may be evaluated in this article, or claim that may be made by its manufacturer, is not guaranteed or endorsed by the publisher.

## Footnotes

^1^ http://www.bioinformatics.babraham.ac.uk/projects/fastqc
