# Corrigendum: Polymerase II–associated factor 1 complex-regulated *FLOWERING LOCUS C*-Clade genes repress flowering in response to chilling

**DOI:** 10.3389/fpls.2023.1346245

**Published:** 2024-01-09

**Authors:** Zeeshan Nasim, Hendry Susila, Suhyun Jin, Geummin Youn, Ji Hoon Ahn

**Affiliations:** Department of Life Sciences, Korea University, Seoul, Republic of Korea

**Keywords:** *Arabidopsis*, FLC, MAFs, PAF1C, flowering, epigenetics, temperature

In the published article, there was an error in Supplementary Table 1, sub-section “qPCR primers”. The sequences of two qPCR primers were incorrectly submitted. The primer sequence of *MAF2* and *MAF4* in the current table are:

**Table:** 

*MAF2*	Sense	GCCACAGCCCACCGGAGAC
	Antisense	TCAGCCGTTGATGATTGGTGGT
*MAF4*	Sense	TCTGCCACCGGAAGACTCTA
	Antisense	CGGTGACATTGCTCTTGCAT

The correct sequences of the primers used in the study are:

**Table:** 

*MAF2*	Sense	AGCTCGAGACTGCTCTGTCC
	Antisense	CCATTTTCCCATGACATTCC
*MAF4*	Sense	TCGCACAAGGAGTTGCTAGA
	Antisense	GGGCTTCACAAGCTCCATC

The authors apologize for this error and state that this does not change the scientific conclusions of the article in any way.

