# Supplementary material for: Polymerase II–Associated Factor 1 Complex-Regulated FLOWERING LOCUS C-Clade Genes Repress Flowering in Response to Chilling
Source: Front Plant Sci. 2022 Feb 9;13:817356. doi: 10.3389/fpls.2022.817356 (PMC8863679; doi:10.3389/fpls.2022.817356)
Supplement: Supplementary file 1 [file Data_Sheet_1.docx]

Supplementary Material

PAF1c-regulated *FLC*-clade genes are important for the repression of reproductive transition in response to chilling temperature

Zeeshan Nasim, Hendry Susila, Suhyun Jin, Geummin Youn, and Ji Hoon Ahn ^*^

# * Correspondence: Ji Hoon Ahn: jahn@korea.ac.kr

# Supplementary Data

# Supplementary Figures and Tables

## Supplementary Figures


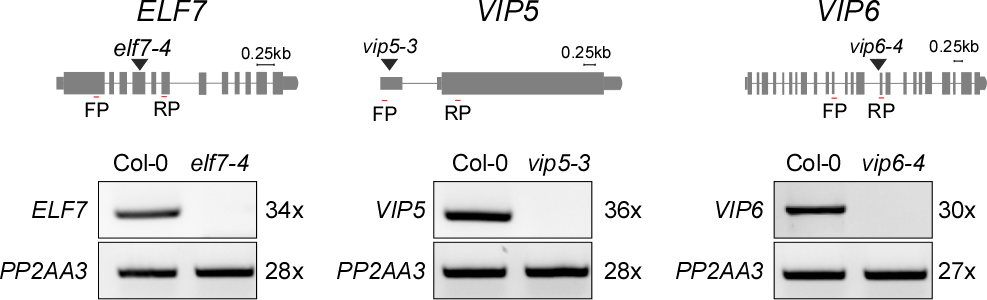


**Supplementary Figure 1**. Schematic representation of the T-DNA insertion sites in previously uncharacterized *elf7-4*, *vip5-3*, and *vip6-5* alleles and the position of primers used for RT-PCR (upper panels). *PP2AA3* was used as an internal loading control. FP, forward primer; RP, reverse primer. Numbers to the right of the gel images represent the number of PCR cycles. Scale bar = 0.25 kb.


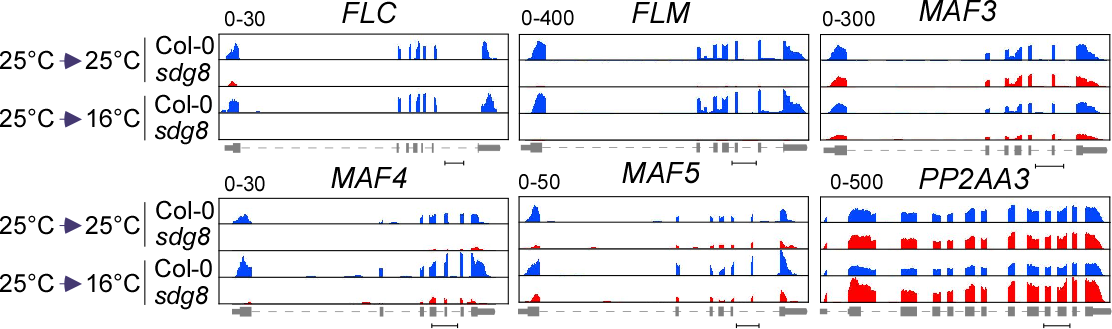


**Supplementary Figure 2:** Expression of *FLC* and *FLC*-clade genes in *sdg8* mutants at different temperature conditions, derived from public RNA-seq data (GSE8528). Scale bar = 0.4 kb.


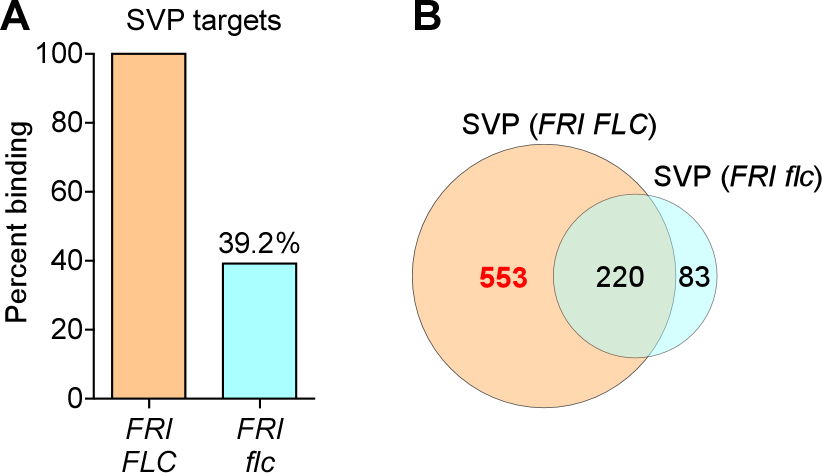


**Supplementary Figure 3:** SVP-GFP binding to downstream targets requires FLC. **(A)** Difference in the percent target binding of SVP-GFP with and without FLC. SVP-GFP target binding with functional FLC was set as 100%. A publicly available SVP-GFP ChIP-seq dataset (GSE54881) was used. **(B)** Venn diagram showing the number of SVP-bound targets in seedlings with or without functional FLC.

## Supplementary Tables

**Supplementary Table 1.** Details of the primers used in this study

| **Genotyping primers** | | | |
| --- | --- | --- | --- |
| **Gene** | **T-DNA line** | **Orientation** | **Sequence** |
| *ELF7* | SALK_ 046605 | LP | ATCGAGACCGAAGAAGAGAGG |
|  |  | RP | TTGCCTGATTCGGTATTTCAG |
|  | SALK_070632 | LP | TAAACCTACATCCAAAGCCCC |
|  |  | RP | TGACACAGGTACGATGAGCAG |
| *PHP* | SALK_150644 | LP | ATCGAAAGCCAAGATGTAGCC |
|  |  | RP | CAACCTCCATATCTGCAATCG |
|  | SALK_008357 | LP | GGTGTCAAATCTCTCACCGAG |
|  |  | RP | GACTGCGTACCGATCAAAAAG |
| *VIP3* | SALK_083364 | LP | ACAGAGAGACCACGAGAGCAG |
|  |  | RP | GAAGCAAATAAAAACTCCACTGC |
|  | SALK_060207 | LP | GAACAGCTTCAACGCAAGTTC |
|  |  | RP | AAGGAGGAGCTTCCAAAACAG |
| *VIP4* | SALK_122755 | LP | GTTGTTGAGAGCGGATCAGAG |
|  |  | RP | TTCTTCAACGAATGTTTTGGC |
|  | SALK_006392 | LP | GGGGAGGTCTTCAATGACTTC |
|  |  | RP | GAGAGTTTCGTTCACTGGTCG |
| *VIP5* | SALK_062223 | LP | GTCTCTTGCCATCGTTCTCTG |
|  |  | RP | ATGTGCCTACGTTTGAGGATG |
|  | SALK_055889 | LP | TCAGAATCATCCCTTGAATCG |
|  |  | RP | TTAGACATGCCGTTAAGTGGG |
| *VIP6* | SALK_065364 | LP | AATCCCCATTGGACGAATTAG |
|  |  | RP | GGGGAACTGGAACTATTTTGC |
|  | SALK_119910 | LP | CCCCTAAAACATTCCAGGAAG |
|  |  | RP | AAAACTTGAGGAGCCGTTTTC |
| **RT-PCR primers** | | | |
| **Gene** | | **Orientation** | **Sequence** |
| *ELF7* | | Sense | TCTCAGATGCCCAAGGGACAC |
|  |  | Antisense | GGATGCTTCAATATCCTTGATTTGT |
| *VIP5* | | Sense | AGCATCTGCTTCCTCTCGAC |
|  |  | Antisense | ATTCCAGCAAGCTTCTGCCT |
| *VIP6* | | Sense | AGTACATGCGAAAGGCCACA |
|  |  | Antisense | ACCAGTTCAATGGCCAGAGG |
| **qPCR primers** | | | |
| **Gene** | | **Orientation** | **Sequence** |
| *FLC* | | Sense | TGTGAGTATCGATGCTCTTGTTCA |
|  |  | Antisense | TTCAACATGAGTTCGGTCTTCTTG |
| *FLM*-*beta* | | Sense | CGCAATGGTCTCATCGACAAAG |
| *FLM*-common | | Antisense | CAGCAACGTATTCTTTCCCAT |
| *FLM*-*delta* | | Sense | GATAGAAGCGCTGTTCAAGC |
| *FT* | | Sense | AGGCCTTCTCAGGTTCAAAACAAGC |
|  |  | Antisense | TGCCAAAGGTTGTTCCAGTTGTAGC |
| *MAF2* | | Sense | AGCTCGAGACTGCTCTGTCC |
|  |  | Antisense | CCATTTTCCCATGACATTCC |
| *MAF3* | | Sense | AGCGAATCGAGAACAAAAGC |
|  |  | Antisense | CGGAGGCAGAGTCGTAGAGT |
| *MAF4* | | Sense | TCGCACAAGGAGTTGCTAGA |
|  |  | Antisense | GGGCTTCACAAGCTCCATC |
| *MAF5* | | Sense | TCAGGATCTCCGACCAGTTT |
|  |  | Antisense | CGAGCTTTCTCCATGAGACC |
| *PP2A* | | Sense | GCGGTTGTGGAGAACATGATACG |
|  |  | Antisense | GAACCAAACACAATTCGTTGCTG |
| *SAND* | | Sense | TTGATCCACTTGCAGACAAGGC |
|  |  | Antisense | TACCCTTTGGCACACCTGATTG |
| *SOC1* | | Sense | TGAGGGGCAAAACTCAGATG |
|  |  | Antisense | TCTTGCATATTGGAGCTGGC |
| *SVP* | | Sense | ATCAAGACCTGACTCATTGG |
|  |  | Antisense | AGCATACTCACGACGAAACT |
| **ChIP-qPCR primers**  Sense  GCGTCCGGTGATGTTGAGTA | | | |
| **Gene** | **Position** | **Orientation** | **Sequence** |
| *FLC* | P1 | Sense | CGTGAGTCCGCCCCTGATAGC |
|  |  | Antisense | GGACCAAACCAAACCTACAAAGACTTTC |
|  | P2 | Sense | CTTAGTATCTCCGGCGACTTGAACC |
|  |  | Antisense | GCGTCACAGAGAACAGAAAGCTGA |
|  | P3 | Sense | GGCGGATCTCTTGTTGTTTC |
|  |  | Antisense | CTTCTTCACGACATTGTTCTTCC |
|  | P4 | Sense | ACACAACCTTTGTATCTTGTGTCTTTTG |
|  |  | Antisense | AGTAGACACTACACCAGATTCAATTTTGAC |
| *FLM* | P1 | Sense | GTCGTGGGCTGATTCTGGTTCACC |
|  |  | Antisense | CCATTACTTTATCGAGTTAGGTCGTAGGTTCT |
|  | P2 | Sense | CTTATCGGAGATTTGAAGCCATGGGAAGAAG |
|  |  | Antisense | GGATTCACAGAGAATCGAAAGTTGTCGAGC |
|  | P3 | Sense | CAGTATGTGTATATATACAGCTCCTTCTCATTTCCT |
|  |  | Antisense | GAGACAGATAGATACGGTTCCTAATACTTCGTC |
|  | P4 | Sense | CCGTAAGTAGAGCTAGGAAGGTATATGTGCTGC |
|  |  | Antisense | GACTCGATATACTCCATCATCAGTTCTGCCTATAAC |
| *MAF2* | P1 | Sense | AACTTTGAACTTAAATCTTGTTTTTTTTGGTGACAGCAC |
|  |  | Antisense | TTCAAACCTTCACTAAACTTATACGGATGGCC |
|  | P2 | Sense | TCCGGTGACAAGTAAGCTGCTACTTTCC |
|  |  | Antisense | GTTGATTTGAAGGAAACAAAAAGCCCTAGATTTG |
|  | P3 | Sense | GATATCGAGCATCGATTTCTACCGATAGGGTG |
|  |  | Antisense | TAAAAGAGAAGTAGTAGTTGAGATGTCTTCAAGATAGACG |
|  | P4 | Sense | CCTTATGATCTTGTCCAAACAGGAGAACTTGCTG |
|  |  | Antisense | CAAAAAAAATGCCTAACATCAATCAAAGTCCTTCAAC |
| *MAF3* | P1 | Sense | GTTATTTGATTACTTGCACGTGACGTGGCATAGTG |
|  |  | Antisense | GCAGAAAAATACTTGTCTTGGCTGTGCTTCCAG |
|  | P2 | Sense | GACGCAAAGGTCTCATCGAAAAAGCTCG |
|  |  | Antisense | GAAAGAAGCTTACTTGTCACCGGAGGC |
|  | P3 | Sense | CATTTCTTCAACTTTGGTTGTGGATATTCCTAACCAC |
|  |  | Antisense | TTTGTCAATGTAGGTTAATTTCGTTTGGTTCAGTG |
|  | P4 | Sense | TGACAGAGGAATGTCACGGGAAAATGG |
|  |  | Antisense | TTTGTAAAAGCTTCTGAATCAGGCTGTGAGTAAG |
| *MAF4* | P1 | Sense | GGCAGAAAAAGACTTGTCGAGGCTGG |
|  |  | Antisense | GTGTGTTTTCCAAAAATCCAAAAATTTGAACCGAG |
|  | P2 | Sense | CAAACGAATTGAGAACAAAAGCTCTCGACAAGT |
|  |  | Antisense | GCTGTAGAGTCTTCCGGTGGCAGA |
|  | P3 | Sense | CAGTGGAAGAAAATAGCATGTGTACACATTGAGTAGC |
|  |  | Antisense | CGTACCAAATGTTTGTAGAGACTCAGTTTCTTTGG |
|  | P4 | Sense | TTTCTTACAGATGGGGAAGATGAAGAAGTCTG |
|  |  | Antisense | GGTGATGGTGGTTACTTGAGAAGCAGGAG |
| *MAF5* | P1 | Sense | CATGAAATTATTGGATGACTCTTCTTCCCCTAAGC |
|  |  | Antisense | ACAAACTACTGCTCAAGTTATTCCAGCTCAGG |
|  | P2 | Sense | AGCAGTAGACAAGTCACTTTCTGTAAGAGACG |
|  |  | Antisense | GAAGAGACGATGAAAAGAGCGACGGAG |
|  | P3 | Sense | GTCGTTTTGGAGATATTGGGTCTCATGGG |
|  |  | Antisense | CAAGGACATCCCCATTTCTACATTATAGAGGG |
|  | P4 | Sense | GGGGATGAAAGAGCAGTAATGTCACCG |
|  |  | Antisense | TGAATCAGGATGATGAAAAATCAGCCGTTGATG |
| **Cloning primers** | | | |
| **Clone** | **Primer** | **Vector** | **Sequence** |
| *35S::amiR-*  *MAF2-5-*  1^st^ | I miR-s | *pENTR2B* | gaTAATTGAGAGTTGACGAGCTGtctctcttttgtattcc |
|  | II miR-a |  | gaCAGCTCGTCAACTCTCAATTAtcaaagagaatcaatga |
|  | III miR*s |  | gaCAACTCGTCAACTGTCAATTTtcacaggtcgtgatatg |
|  | IV miR*a |  | gaAAATTGACAGTTGACGAGTTGtctacatatatattcct |
| *35S::amiR-*  *MAF2-5-*  2^nd^ | I miR-s | *pENTR2B* | gaTAATTGAGAGTTGACGAGCTTtctctcttttgtattcc |
|  | II miR-a |  | gaAAGCTCGTCAACTCTCAATTAtcaaagagaatcaatga |
|  | III miR*s |  | gaAAACTCGTCAACTGTCAATTTtcacaggtcgtgatatg |
|  | IV miR*a |  | gaAAATTGACAGTTGACGAGTTTtctacatatatattcct |
| *BD-MAF2* | FP | *pGBKT7* | ccatggaggccgaattccccATGGGTAGAAAAAAGT |
|  | RP |  | ctgcaggtcgacggatccccCTTGAGCAGCGGAAGAGTCTCCC |
| *BD-MAF3* | FP | *pGBKT7* | ccatggaggccgaattccccATGGGAAGAAGAAAAGT |
|  | RP |  | ctgcaggtcgacggatcccctcaCTTGAGCAGCGAAAGAGTCTCCG |
| *BD-MAF4* | FP | *pGBKT7* | ccatggaggccgaattccccATGGGAAGAAGAAAAGT |
|  | RP |  | ctgcaggtcgacggatcccctcaGAGAAGCAGGAGAGTCTCCGGTG |
| *BD-MAF5* | FP | *pGBKT7* | ccatggaggccgaattccccATGCTTGAAGACAAAAC |
|  | RP |  | ctgcaggtcgacggatcccctcaGAGAAGCGGGAGAGTCTCCGGTG |
| *AD-SVP* | FP | *pGADT7* | aggccagtgaattccaccccATGGCGAGAGAAAAGATTCA |
|  | RP |  | tcccgtatcgatgcccaccctcaACCACCATACGGTAAGCC |
| *35S::MAF2-GFP* | FP | *p326-GFP* | ttcatttggagagaacacgtctagaATGGGTAGAAAAAAAGT |
|  | RP |  | actcattgttatatctccttggatccgCTTGAGCAGCGGAAGAGTCTCCC |
| *35S::MAF3-GFP* | FP | *p326-GFP* | ttcatttggagagaacacgtctagaATGGGAAGAAGAAAAGT |
|  | RP |  | actcattgttatatctccttggatccgCTTGAGCAGCGAAAGAGTCTCCG |
| *35S::MAF4-GFP* | FP | *p326-GFP* | ttcatttggagagaacacgtctagaATGGGAAGAAGAAAAGT |
|  | RP |  | actcattgttatatctccttggatccgGAGAAGCAGGAGAGTCTCCGGTG |
| *35S::MAF5-GFP* | FP | *p326-GFP* | ttcatttggagagaacacgtctagaATGCTTGAAGACAAAAC |
|  | RP |  | actcattgttatatctccttggatccgGAGAAGCGGGAGAGTCTCCGGTG |

For cloning primers: uppercase letters = Coding sequences, lowercase letters = infusion/restriction enzyme sites

**Supplementary Table 2.** Flowering time of PAF1C-deficient mutants across a broad temperature range (10°C – 27°C) under longday (LD) conditions.

| **Genotype** | **TLN** | **TLN StDev** | **TLN range** | ***n*** |
| --- | --- | --- | --- | --- |
| **10°C LD** |  |  |  |  |
| Col-0 | 36.3 | 1.8 | 34-39 | 11 |
| *elf7-2* (SALK_ 046605) | 17.0 | 1.5 | 14-19 | 13 |
| *elf7-4* (SALK_070632) | 19.9 | 1.1 | 18-22 | 12 |
| *cdc73-1* (SALK_150644) | 25.1 | 1.2 | 23-27 | 12 |
| *cdc73-2* (SALK_008357) | 27.2 | 0.9 | 26-28 | 12 |
| *vip3-2* (SALK_083364) | 14.3 | 1.9 | 12-16 | 12 |
| *vip3-6* (SALK_060207) | 14.7 | 1.0 | 13-16 | 12 |
| *vip4-1* (SALK_122755) | 16.8 | 1.2 | 14-18 | 12 |
| *vip4-3* (SALK_006392) | 18.4 | 0.7 | 17-19 | 11 |
| *vip5-2* (SALK_062223) | 14.5 | 1.4 | 12-16 | 11 |
| *vip5-3* (SALK_055889) | 15.6 | 1.3 | 14-18 | 11 |
| *vip6-2* (SALK_065364) | 15.1 | 1.2 | 13-17 | 14 |
| *vip6-5* (SALK_119910) | 15.6 | 1.4 | 13-18 | 12 |
| **16°C LD** | | | | |
| Col-0 | 32.1 | 2.3 | 28-34 | 12 |
| *elf7-2* (SALK_ 046605) | 14.1 | 0.9 | 13-16 | 13 |
| *elf7-4* (SALK_070632) | 15.1 | 0.7 | 14-16 | 10 |
| *cdc73-1* (SALK_150644) | 21.7 | 2.1 | 18-25 | 17 |
| *cdc73-2* (SALK_008357) | 27.2 | 1.3 | 26-28 | 17 |
| *vip3-2* (SALK_083364) | 12.0 | 0.8 | 10-13 | 26 |
| *vip3-6* (SALK_060207) | 12.2 | 1.0 | 10-14 | 23 |
| *vip4-1* (SALK_122755) | 14.2 | 1.0 | 13-16 | 14 |
| *vip4-3* (SALK_006392) | 14.5 | 0.9 | 13-16 | 25 |
| *vip5-2* (SALK_062223) | 12.4 | 1.1 | 11-14 | 24 |
| *vip5-3* (SALK_055889) | 13.1 | 1.6 | 11-14 | 12 |
| *vip6-2* (SALK_065364) | 12.5 | 0.9 | 11-14 | 17 |
| *vip6-5* (SALK_119910) | 12.4 | 1.0 | 10-14 | 20 |
| **23°C LD** | | | | |
| Col-0 | 15.6 | 0.9 | 14-17 | 14 |
| *elf7-2* (SALK_ 046605) | 10.0 | 0.8 | 9-11 | 13 |
| *elf7-4* (SALK_070632) | 10.1 | 0.9 | 10-12 | 11 |
| *cdc73-1* (SALK_150644) | 12.2 | 0.8 | 11-14 | 21 |
| *cdc73-2* (SALK_008357) | 12.1 | 0.8 | 10-13 | 17 |
| *vip3-2* (SALK_083364) | 8.8 | 0.6 | 8-10 | 19 |
| *vip3-6* (SALK_060207) | 9.0 | 0.8 | 8-10 | 17 |
| *vip4-1* (SALK_122755) | 10.5 | 0.9 | 9-12 | 15 |
| *vip4-3* (SALK_006392) | 10.3 | 0.7 | 10-12 | 22 |
| *vip5-2* (SALK_062223) | 9.2 | 1.0 | 8-11 | 17 |
| *vip5-3* (SALK_055889) | 9.6 | 1.7 | 8-11 | 14 |
| *vip6-2* (SALK_065364) | 8.8 | 0.7 | 8-10 | 14 |
| *vip6-5* (SALK_119910) | 9.3 | 0.9 | 8-11 | 20 |
| **27°C LD** |  |  |  |  |
| Col-0 | 12.5 | 0.9 | 11-14 | 20 |
| *elf7-2* (SALK_ 046605) | 9.1 | 0.9 | 8-11 | 13 |
| *elf7-4* (SALK_070632) | 11.0 | 1.0 | 10-13 | 10 |
| *cdc73-1* (SALK_150644) | 10.1 | 0.7 | 9-11 | 21 |
| *cdc73-2* (SALK_008357) | 10.5 | 0.6 | 9-11 | 21 |
| *vip3-2* (SALK_083364) | 9.0 | 0.7 | 8-11 | 21 |
| *vip3-6* (SALK_060207) | 9.2 | 0.5 | 8-10 | 19 |
| *vip4-1* (SALK_122755) | 8.2 | 0.8 | 8-10 | 15 |
| *vip4-3* (SALK_006392) | 9.5 | 1.1 | 8-11 | 12 |
| *vip5-2* (SALK_062223) | 9.2 | 0.8 | 8-10 | 20 |
| *vip5-3* (SALK_055889) | 9.5 | 1.0 | 8-11 | 15 |
| *vip6-2* (SALK_065364) | 9.6 | 1.0 | 8-11 | 14 |
| *vip6-5* (SALK_119910) | 9.8 | 0.9 | 8-11 | 16 |

**Supplementary Table 3.** Leaf number ratio (LNR) of PAF1C-deficient mutants across at different temperatures, under LD conditions.

| **Genotype** | **Mean LNR** | | |
| --- | --- | --- | --- |
|  | 16°C/23°C | 10°C/23°C | 23°C/27°C |
| Col-0 | 2.05 | 2.32 | 1.25 |
| *elf7* | 1.41 | 1.71 | 1.10 |
| *php* | 1.77 | 2.23 | 1.18 |
| *vip3* | 1.36 | 1.62 | 0.98 |
| *vip4* | 1.37 | 1.64 | 1.19 |
| *vip5* | 1.36 | 1.59 | 1.01 |
| *vip6* | 1.42 | 1.69 | 0.92 |
